# Interferon score is increased in incomplete systemic lupus erythematosus and correlates with myxovirus-resistance protein A in blood and skin

**DOI:** 10.1186/s13075-019-2034-4

**Published:** 2019-12-02

**Authors:** Wietske M. Lambers, Karina de Leeuw, Berber Doornbos-van der Meer, Gilles F.H. Diercks, Hendrika Bootsma, Johanna Westra

**Affiliations:** 1 Department of Rheumatology and Clinical Immunology, University of Groningen, University Medical Centre Groningen, AA21, Hanzeplein 1, 9700 RB Groningen, the Netherlands; 2 Department of Pathology and Medical Biology, University of Groningen, University Medical Centre Groningen, Groningen, the Netherlands

**Keywords:** Systemic lupus erythematosus, Interferon signature, Myxovirus resistance protein A, Biomarker

## Abstract

**Objectives:**

Patients with incomplete systemic lupus erythematosus (iSLE) have lupus features, but do not meet classification criteria for SLE. Type I interferons (IFN) are important early mediators in SLE, and IFN upregulation in incomplete SLE may be associated with progression to SLE. Since many patients present with skin symptoms, the aim of this study is to investigate IFN type I expression and IFN-related mediators in the blood and skin of iSLE patients.

**Methods:**

Twenty-nine iSLE patients (ANA titer ≥ 1:80, symptoms < 5 years, ≥ 1 objectified clinical criterion), 39 SLE patients with quiescent disease (fulfilling ACR or SLICC criteria, SLEDAI ≤4), and 22 healthy controls were included. IFN signature was measured in whole blood, based on 12 IFN-related genes, using RT-PCR, and IFN-score was calculated. IFN-related mediators myxovirus-resistance protein A (MxA), IFN-γ-induced protein 10 (IP-10), and monocyte chemoattractant protein (MCP-1) were measured using ELISA. IFN type I expression in the unaffected skin was analyzed by immunostaining with MxA.

**Results:**

IFN-score was increased in 50% of iSLE patients and 46% of SLE patients and correlated positively with the number of autoantibodies, anti-SSA titer, ESR, and IgG and negatively with C4 in iSLE. Levels of MxA correlated strongly with IFN-score (*r* = 0.78, *p* < 0.0001). Furthermore, MxA expression was found in 29% of unaffected skin biopsies of iSLE and 31% of SLE patients and also correlated with IFN-score (*r* = 0.54, *p* < 0.0001).

**Conclusions:**

IFN-score was increased in half of the iSLE patients, and given the correlation with complement and autoantibody diversity, this suggests a higher risk for disease progression. MxA in the blood and unaffected skin correlated strongly with the IFN-score and is possibly an easily applicable marker for IFN upregulation.

## Introduction

Systemic lupus erythematosus (SLE) is a prototypic autoimmune disease that is characterized by formation of antinuclear antibodies (ANA) and inflammation of various organ systems [1]. Despite the progress in understanding the pathogenesis of SLE and the subsequent increase in treatment options, disease burden is still high [2]. A frequent diagnostic delay, attributable to the heterogeneous clinical picture, is one of the main reasons for damage accrual [3].

Although clinical expert opinion is still the gold standard for SLE diagnosis, classification criteria have been developed for scientific purposes. The criteria developed by the American College of Rheumatology (ACR) and updated in 1997 are most widely used [4, 5]. In 2012, new classification criteria were introduced by the Systemic Lupus International Collaborating Clinics (SLICC) group in order to improve clinical relevance and to incorporate new knowledge regarding the pathogenesis of SLE [6]. Very recently, new EULAR/ACR classification criteria for SLE have been published [7].

A subgroup of lupus patients displays clinical symptoms or immunologic abnormalities that are typical for SLE, but do not fulfill the classification criteria. This condition is referred to as “incomplete systemic lupus erythematosus” (iSLE). Some of these patients remain in a clinically stable state of limited disease, but 10–55% develop SLE [8–11]. Investigating incomplete SLE is of interest, since it could provide insight in disease pathogenesis and possibly reveal predictive biomarkers for SLE. Consequently, stratifying the risk for disease progression will allow less frequent follow-up for low-risk patients, while on the other hand high-risk patients can be monitored more closely, and possibly start treatment earlier.

Although the precise pathophysiology of SLE is complex and has not been fully elucidated yet, IFN-alpha, which is a type I IFN, has been demonstrated to be an important early mediator [12]. Last year, Yusof et al. showed that IFN gene upregulation in patients with ANA positivity and at least one SLICC criterion was associated with progression to SLE [13]. Plasmacytoid dendritic cells (pDCs) are the most potent producers of IFN-alpha and respond to binding of RNA- and DNA-containing autoimmune complexes to Fcγ receptors on the cell surface. Subsequent activation of endosomal toll-like receptors (TLRs) 7 and -9 results in the production of large amounts of IFN-alpha [14]. This cytokine supports differentiation, proliferation, and survival of T and B cells and hence enhances production of more autoantibodies resulting in a feed-forward loop [15–17].

As it is rather difficult to measure IFN-alpha in serum due to low levels and short lifetime, expression of IFN-regulated genes is usually measured instead. Most SLE patients show increased expression of IFN-regulated genes in peripheral blood mononuclear cells or whole blood, which is referred to as IFN signature [12, 18]. Following modular transcript analysis, IFN-regulated genes are distributed over three gene modules (M1.2, M3.4, and M5.12) [19]. Module 1.2 is mainly induced by IFN type I and is stable over time, while the latter two—that are sequentially activated—are induced by both IFN type I and II and were previously shown to correlate with disease activity longitudinally [20].

As measuring IFN signature is time consuming, there is a need for easily applicable markers for IFN expression. Myxovirus-resistance protein A (MxA) is strictly induced by IFN type I and a recent study showed strong robust correlation with IFN gene upregulation [21]. Furthermore, interferon-inducible chemokines IFN-γ-induced protein 10 (IP-10) and monocyte chemoattractant protein 1 (MCP-1) could be suitable as surrogate markers [22].

Likely, initiation of autoimmunity takes place in tissue and not only in the circulation. Therefore, it was decided to additionally analyze MxA in the skin. MxA is already known to be expressed in active lupus skin lesions [23]. Skin is accessible for biopsy and relevant as up to 80% of SLE patients will develop skin symptoms. Furthermore, IFN type I plays a role in the formation of lupus skin lesions [24, 25].

The aim of the current study is to assess IFN gene expression and levels of IFN-related mediators in both circulation and skin in iSLE patients in relation to HC and SLE.

## Materials and methods

### Study population

Patients with iSLE were identified by screening the diagnosis registration system of our tertiary referral center for “incomplete lupus,” “cutaneous lupus,” and “immune disease not specified.” iSLE was defined as (1) ANA-titer ≥ 1:80; (2) one other objective clinical ACR criterion for SLE, but not meeting ACR criteria or SLICC criteria; and (3) disease manifestations < 5 years. Use of hydroxychloroquine (HCQ) was allowed, but iSLE patients using steroids or other immunosuppressants were excluded in order to avoid treatment effects. SLE patients with clinically quiescent disease (SLE daily activity index (SLEDAI) score ≤ 4) were selected as positive controls. All SLE patients fulfilled ACR or SLICC criteria, and all had disease duration < 10 years and the use of immunosuppressants in this patient group was allowed. During the out-patient visit, additional blood was withdrawn and a skin biopsy was taken. Results were compared to healthy controls (HC), matched for age and gender with a ratio of 3:1 (iSLE versus HC).

The research protocol was approved by the Institutional Medical Ethical Committee (METc 2015/313). All subjects provided written informed consent.

### Laboratory investigations

All clinical data and standard laboratorial measurements were retrieved from medical records. Antinuclear antibodies (ANA) were measured by indirect immunofluorescence technique using Hep2-cells as substrate. Anti-double-stranded DNA antibodies (anti-dsDNA) and other autoantibodies were measured using the automated EliA assay (ENA CTD screen, Thermo Fisher Scientific, Nieuwegein, the Netherlands).

#### Interferon signature

Whole blood samples were collected in PAXgene RNA tubes and stored at − 20 °C. After thawing, RNA was isolated using PAXgene Blood RNA Kit (Cat No./ID:762164).

Isolated RNA was reversely transcribed to cDNA and subsequently quantitatively analyzed using an Applied Biosystems™ QuantStudio™ 6 Flex Real-Time PCR System. Relative expression (RE) was calculated based on the cycle threshold (Ct) value related to expression of the housekeeping gene GAPDH as follows: RE = 2^-(Ct Test gene − Ct GAPDH)^. The Ct value of GAPDH was confirmed to be stable in all performed analyses in all cohorts.

Twelve IFN-related transcripts were determined, representing all three IFN modules (M1.2: IP10, IFI44L, IFIT3, LY6E, MX1, and SERPING1; M3.4: IFITM1, IRF7, and STAT1; M5.12: C1QA, IFI16, and IRF9) [20]. In order to construct a workable unity to compare gene expression between individuals, an IFN-score was generated. The IFN-score was calculated by summing up the individual RE per gene after normalization to the control group as follows: ∑(RE_subject_ − Mean_hc_)/SD_hc_ [26]. Regarding the three interferon modules, a separate gene score was calculated, based on the genes from the concerning module (M1.2, M3.4, and M5.12). In order to increase comparability with other studies, an IFN3-score was composed, based on the sum of normalized RE of three widely used IFN genes (IFI44L, LY6E, and MX1). An IFN-score was regarded positive when it was higher than the mean + 2SD of HC values. In one iSLE patient, Paxgene tubes were not available; therefore, this patient could not be included in IFN gene measurements.

#### IFN-related mediators

Levels of serum IP-10 and MCP-1 were measured by ELISA (Duoset, R&Dsystems, Minneapolis, Canada). High-performance ELISA buffer (Sanquin, Amsterdam, the Netherlands) was used during serum incubation to prevent non-specific reactions.

Levels of MxA were measured by ELISA in lysed whole blood as described previously [27]. Intra- and inter-assay variation of this ELISA was 7% and 6%, respectively.

Cut-off levels were determined based on the mean + 2SD of the control group, resulting in the values of 94.5 pg/mL for IP-10, 391.2 pg/mL for MCP-1, and 55.9 ng/mL for MxA.

### Skin biopsy analysis

Subjects underwent biopsy of 4-mm unaffected and non-sunexposed skin from the buttock. Skin biopsies were formalin-fixed and embedded in paraffin.

Sections were deparaffinized, and antigen retrieval was performed with tris-HCl and EDTA. Goat-anti human MxA (R&D, AF7946, Minneapolis, Canada) was added overnight; consecutively, samples were incubated with rabbit anti-goat-HRP conjugate (Dako, 0449, Santa Clara, USA) and stained with diaminobenzidine (DAB) colorant (Dako K4006, Santa Clara, USA).

Control staining with the addition of secondary antibody alone and using normal goat serum was negative. A positive control of the lesional lupus skin showed strong MxA expression throughout all dermal structures (see Additional file 1: Figure S1). Scoring of MXA expression was done by a team of a pathologist and two investigators. The samples were blinded. The expression of MxA was scored semi-quantitatively for separate structures, namely the epidermis, endothelium, adnexa, fibroblasts, and infiltrates, as follows: “0” for no expression, “0.5” for weak expression, “1” for moderate expression, “2” for intermediate expression, and “3” for strong expression.

#### Statistical analysis

Comparison between groups was performed using Mann-Whitney *U* test for continuous data and chi-square test for dichotomous variables. Correlations were calculated using Spearman *r* test. Correlations and group comparison were calculated for a minimum of 5 subjects. Analyses were carried out using IBM SPSS Statistics version 23, and images were created using GraphPad Prism 7.02.

## Results

### Characteristics

Patient characteristics and cumulative disease characteristics are shown in Table 1. The vast majority of patients were Caucasian. Median age of iSLE and SLE patients was 43 and 44 years respectively. As expected, iSLE disease duration was shorter (1.4 versus 2.8 years). Current disease features and laboratory measurements are shown in Table 2.

**Table 1:** Baseline characteristics

	HC (*n* = 22)	iSLE (*n* = 29)	SLE (*n* = 39)	*p* value
Female gender, *n* (%)	18 (82)	23 (79)	32 (82)	NS
Age (years)	45 (24–65)	44 (20–83)	43 (19–76)	NS
Race, *n* (%)				
Caucasian	100	25 (86)	35 (90)	
Asian	0	2 (7)	1 (3)	
Other	0	2 (7)	3 (8)	
Disease duration (years)		1.4 (0.1–4.6)	2.8 (0.5–6.8)	*0.004*
ACR criteria		3 (1–3)	5 (2–9)	*< 0.0001*
SLICC criteria		3 (2–4)*	5 (3–9)	*< 0.0001*
Clinical criteria, *n* (%)				
Skin involvement		13 (45)	21 (54)	0.46
Photosensitivity		3 (10)	9 (23)	0.17
Arthritis		6 (21)	18 (46)	*0.03*
Alopecia		0	5 (13)	0.05
Ulcera		1 (3)	6 (15)	0.11
Hematologic		11 (38)	26 (67)	*0.02*
Serositis		2 (7)	9 (23)	0.07
Renal		0	13 (33)	*0.001*
Neurologic		1 (3)**	2 (5)	0.48
Immunologic criteria, *n* (%)				
ANA	3 (14)	29 (100)	39 (100)	NA
Anti-dsDNA	0	9 (31)	32 (82)	*< 0.0001*
Anti-SSA	0	13 (45)	13 (33)	0.59
Anti-Smith	0	2 (7)	6 (15)	0.28
Decreased complement	2 (9)	4 (14)	25 (64)	*< 0.0001*
Antiphospholipid Ab	0	7 (24)	17 (44)	*0.01*
Coombs positive	NA	4/23 (17)	7/17 (41)	0.10

*One patient had 4 immunologic SLICC criteria, but no clinical criterion

**Small fiber neuropathy not otherwise explained

For continuous data, median (range) are shown. *p* values of nominal variables are calculated for comparison of iSLE and SLE using chi-square test and continuous variables by using Mann-Whitney *U* test. *p* values < 0.05 are indicated by italic font

*Abbreviations: ANA* antinuclear antibody, *anti-dsDNA* anti-double-stranded DNA, *Ab* antibodies, *NS* non-significant, *NA* not available

**Table 2:** Clinical and serological features at the time of inclusion

	HC (*n* = 22)	iSLE (*n* = 29)	SLE (*n* = 39)
SLEDAI (median, range)	NA	0 (0–6)	2 (0–4)
Presence of clinical symptoms (*n*, %)	NA		
Active cutaneous lupus		5 (17)	4 (10)
Photosensitivity		9 (31)	8 (21)
Arthritis		1 (3)	0
Alopecia		0	0
Objectified ulcera		0	1 (3)
Hematologic features		11 (38)	19 (49)
Serositis		0	0
Renal features		0	1 (3)
Neurologic features		2 (7)	0
Laboratory results			
ANA titer	40 (40–160)	160 (40–640*)^•^	80 (40–640*)^•^
Anti-dsDNA positive (*n*, %)	0	8 (28)	16 (41)
Anti-SSA positive (*n*, %)	0	12 (41)	12 (31)
Anti-Smith positive (*n*, %)	0	1 (3)	5 (13)
Anti-U1RNP (*n*, %)	0	2 (7)	5 (13)
Decreased complement (*n*, %)	0	6 (21)	15 (39)
Antiphospolipid antibodies (*n*, %)	0	10 (35)	12 (31)
ESR (mm/h)	5 (1–15)	19 (3–51)^•^	14 (3–96)^•^
Hb (mmol/)	8.20 (7.5–9.1)	8.3 (7.0–9.4)	8.0 (6.2–9.8)
Leukocytes (×10^9^/L)	5.20 (4.5–7.4)	6.0 (2.6–9.2)	5.5 (2.3–11.9)
Lymphocytes (×10^9^/L)	1.8 (1.3–2.9)	1.6 (0.5–3.2)^‡^	1.2 (0.3–3.1)^•^
Thrombocytes (×10^9^/L)	226 (184–363)	241 (135–406)	230 (112–369)
CRP (mg/L)	0.8 (0.3–3.3)	2.4 (0.3–24)^•^	1.7 (0.3–37)
GFR (mL/min)	98 (76–115)	91 (53–121)	91.5 (48–123)
C3 (g/L)	1.05 (0.68–1.23)	1.07 (0.04–1.47)	1.01 (0.54–1.37)
C4 (g/L)	0.16 (0.10–0.28)	0.20 (0.07–0.38)^‡^	0.16 (0.05–0.34)
IgM (g/L)	1.3 (0.40–1.90)	0.95 (0.20–3.20)	0.7 (0.4–5.1)
IgG (g/L)	11.4 (8.9–16.1)	12.7 (7.2–25.4)	11.4 (5.0–19.5)
Drugs, *n* (%)	NA		
Hydroxychloroquine		9 (31)	33 (85)
NSAID		8 (29)	7 (18)
Prednisolone		0	12 (31)
Dose (median, range)		0	7.5 (5–10)
Mycophenolate		0	10 (26)
Azathioprine		0	6 (15)
Methotrexate		0	2 (5)

^•^*p* value < 0.05 comparing with HC

^‡^*p* value < 0.05 comparing with SLE

*ANA titers higher than 640 and SSA titers higher than 240 were not further diluted

For continuous data, median (range) are shown. *p* values of nominal values are calculated using chi-square test and continuous values using Mann-Whitney *U* test

*Abbreviations*: *NA* not applicable, *ANA* antinuclear antibody, *Anti-dsDNA* anti-double-stranded DNA, *SLEDAI* systemic lupus erythematosus disease activity index, *ESR* erythrocyte sedimentation rate, *NSAID* non-steroid anti-inflammatory drug

#### IFN gene expression

**Fig. 1:**
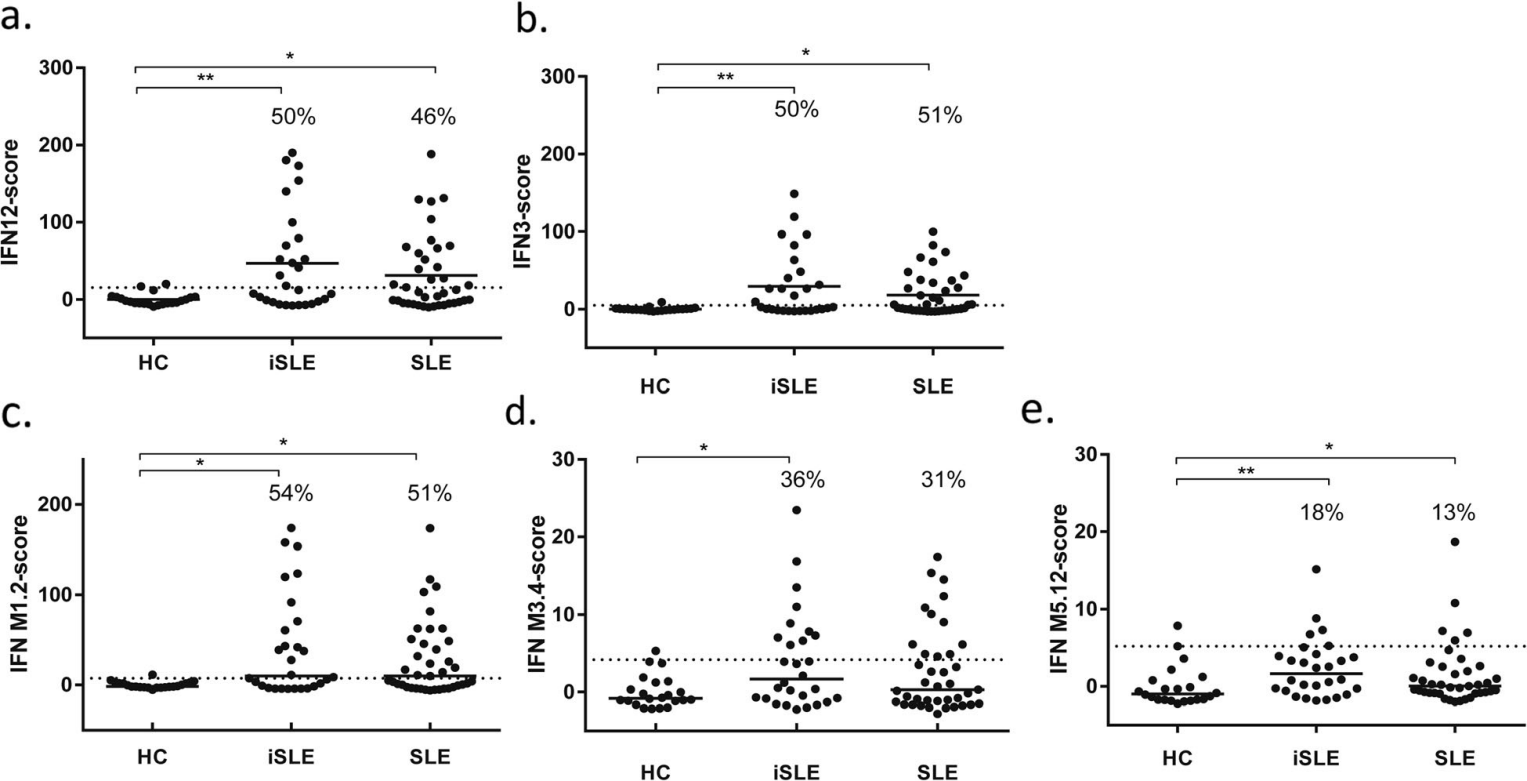
IFN-scores in all subject groups. IFN12-score (**a**), IFN3-score (**b**), and IFN-M1.2, IFN-M3.4, and IFN-M5.12 scores (**c**–**e**). The dotted line represents mean + 2SD of the HC. The percentages represent the proportion of patients above this line. Abbreviations: HC healthy controls, iSLE incomplete systemic lupus erythematosus, SLE systemic lupus erythematosus, IFN interferon, M module

The IFN-score based on 12 genes (IFN12-score) was increased in 14 (50%) iSLE patients and 18 (46%) SLE patients (Fig. 1a).

The patient groups were divided into IFN high and IFN normal according to IFN expression. Characteristics are shown in Table 3. In iSLE, there were no differences regarding clinical symptoms among IFN-high patients and IFN-normal patients. Increased IFN12-score in iSLE patients was not associated with a higher number of cumulative classification criteria, neither with disease duration or SLEDAI. iSLE patients who used HCQ—all patients had therapeutic levels—had similar IFN12-scores as those who did not use this drug. IFN-high iSLE patients however had higher ESR (*p* = 0.004), more antibody diversity (*p* = 0.04), lower C3 (*p* = 0.01), and C4 (*p* = 0.002), as well as increased total IgG (*p* = 0.04).

**Table 3:** Differences across subject groups with normal versus high IFN-score

	iSLE			SLE		
	IFN normal (*n* = 14)	IFN high (*n* = 14)	*p*	IFN normal (*n* = 21)	IFN high (*n* = 18)	*p*
Female gender, *n* (%)	9 (64)	11 (79)	0.40	15 (71)	17 (94)	0.06
Age (years)	50 (25–58)	33 (23–83)	0.10	43 (22–76)	42 (19–74)	0.95
Disease duration (years)	1.9 (0.4–3.4)	1.1 (0.1–4.6)	0.57	2.7 (0.52–6.8)	3.4 (0.7–6.8)	0.61
ACR criteria	3 (1–3)	3 (2–3)	0.70	4 (3–9)	5 (2–8)	0.79
SLICC criteria	3 (2–3)	3 (2–4)	0.70	5 (4–8)	6 (3–9)	0.65
Cumulative criteria						
Clinical, *n* (%)						
Skin involvement	4 (29)	8 (57)	0.13	7 (33)	14 (78)	*0.006*
Photosensitivity	1 (7)	2 (14)	NA	4 (19)	5 (28)	0.52
Arthritis	5 (36)	1 (7)	0.07	9 (43)	9 (50)	0.66
Alopecia	0	0	NA	0 (0)	5 (28)	*0.01*
Ulcera	1 (7)	0 (0)	NA	4 (19)	2 (11)	0.49
Hematologic	5 (36)	5 (36)	0.47	13 (62)	13 (72)	0.50
Serositis	2 (14)	0 (0)	NA	6 (29)	3 (17)	0.38
Renal	0	0	NA	9 (43)	4 (22)	0.17
Neurologic	1 (7)	0 (0)	NA	1 (5)	1 (6)	NA
Immunologic, *n* (%)						
Anti-dsDNA	6 (43)	3 (1)	0.23	17 (81)	15 (83)	0.85
Anti-SSA	5 (36)	8 (57)	0.37	5 (24)	8 (44)	0.18
Anti-Smith	0	2 (14)	NA	1 (5)	5 (28)	*0.05*
Decreased complement	1 (7)	3 (21)	NA	13 (62)	12 (67)	0.47
aPL	3 (21)	4 (29)	0.52	12 (57)	5 (28)	0.18
Medication use						
HCQ	5 (36)	4 (29)	0.67	19 (91)	14 (78)	0.27
NSAIDS	4 (29)	4 (29)	0.90	2 (10)	5 (28)	0.16
Prednisolon				8 (38)	4 (22)	0.28
Azathioprine				5 (24)	1 (6)	0.12
MMF				5 (24)	5 (28)	0.78
SLEDAI	0 (0–6)	1.5 (0–4)	0.40	0 (0–4)	2 (0–4)	*0.03*
Serological values						
ESR (mm/h)	10 (3–51)	25 (6–49)	*0.004*	12 (3–96)	18 (4–57)	0.08
Hb (mmol/)	8.3 (7.0–9.4)	8.0 (7.0–9.4)	0.19	8 (6.2–9.8)	78.0 (7.3–9.4)	0.95
Leukocytes (×10^9^/L)	6.8 (2.6–9.2)	5.6 (2.8–7.5)	0.09	6.8 (2.3–11.9)	5.0 (3.0–7.7)	*0.04*
Lymphocytes (×10^9^/L)	1.7 (0.7–3.2)	1.3 (0.5–2.5)	0.33	1.2 (0.3–3.1)	1.2 (0.7–2.3)	0.84
Monocytes (×10^9^/L)	0.5 (0.4–0.9)	0. 4 (0.2–0.7)	0.06	0.5 (0.3–1.1)	0.5 (0.3–0.7)	0.60
Neutrophils (×10^9^/L)	3.5 (1.5–6.3)	3.4 (1.4–4.9)	0.16	4.1 (1.8–11.0)	2.9 (1.9–5.5)	*0.03*
Thrombocytes (×10^9^/L)	240 (145–406)	238 (135–353)	0.51	238 (120–369)	2413 (112–322)	0.17
CRP (mg/L)	2.5 (0.3–24)	2.5 (0.5–14)	1.0	2.1 (0.3–12)	1.6 (0.3–37)	0.89
GFR (mL/min)	91 (53–107)	92 (80–121)	0.27	91 (48–123)	92 (68–123)	0.90
ANA titer	80 (40–320)	160 (40–640)	0.07	80 (40–640)	160 (40–640)	0.06
Anti-dsDNA (U/mL)	3 (0–50)	1 (0–42)	0.27	5 (0–149)	13 (0–122)	0.26
SSA (U/mL)	0 (0–240)	123 (0–240)	0.09	0 (0–230)	0 (0–240)	0.14
No. of AutoAb	1 (0–1)	1 (0–3)	*0.04*	1 (0–2)	1 (0–5)	0.07
C3 (g/L)	1.2 (0.98–1.47)	1.0 (0.04–1.40)	*0.01*	1.09 (0.74–1.37)	0.91 (0.54–1.32)	*0.03*
C4 (g/L)	0.25 (0.09–0.38)	0.17 (0.07–0.24)	*0.002*	0.17 (0.05–0.34)	0.14 (0.07–0.22)	0.13
IgG (g/L)	11.9 (7.2–14.0)	15.9 (7.7–25.4)	*0.04*	11.4 (5.0–14.4)	11.4 (6.2–19.5)	0.50

Medians with range, and *p* values according to Mann Whitney test are given for continuous values, and numbers with percentages, and *p* values according to chi-square test for dichotomous variables. *p* values < 0.05 are indicated by italic font

*Abbreviations*: *ANA* antinuclear antibody, *Anti-dsDNA* anti-double-stranded DNA, *aPL* antiphospholipid antibodies, *NA* not applicable, *SLEDAI* systemic lupus erythematosus disease activity index, *ESR* erythrocyte sedimentation rate, *HCQ* hydroxychloroquine, *NSAID* non-steroid anti-inflammatory drug, *MMF* mycophenolate mofetil

SLE patients with increased IFN12-scores more often had skin involvement (*p* = 0.006) and alopecia (*p* = 0.01), more frequently had anti-Smith antibodies (*p* = 0.05), and had higher SLEDAI scores (*p* = 0.03). There was no difference in medication use between the IFN-high and IFN-normal SLE patients. Regarding serological tests, IFN-high patients had lower neutrophil counts (*p* = 0.03) and lower C3 levels (*p* = 0.01).

Correlations between IFN12-score and continuous variables revealed no remarkable other findings than when comparing IFN high versus normal (see Additional file 1: Table S2 and Figure S2).

Next, IFN expression was analyzed for each IFN-related module separately (Fig. 1c–e). Among iSLE patients, an increased IFN-M1.2 score was seen in 54%, IFN-M3.4 was increased in 36%, and IFN-M5.12 was increased in 18% (Fig. 1c–e). The modular IFN-scores correlated strongly with each other, as with the overall IFN12-score, with *r* values > 0.8 and *p* values < 0.0001.

At last, the IFN-score based on IFI44L, LY6E, and MX1, 3 commonly used genes (IFN3-score) was calculated. Upregulation was found in 14 (50%) iSLE patients and 20 (51%) SLE patients (Fig. 1b). Correlations were almost identical for IFN3-score compared to IFN12-score (see Additional file 1: Tables S1 and S2).

### Levels of IFN-related soluble mediators and correlations with clinical parameters

**Fig. 2:**
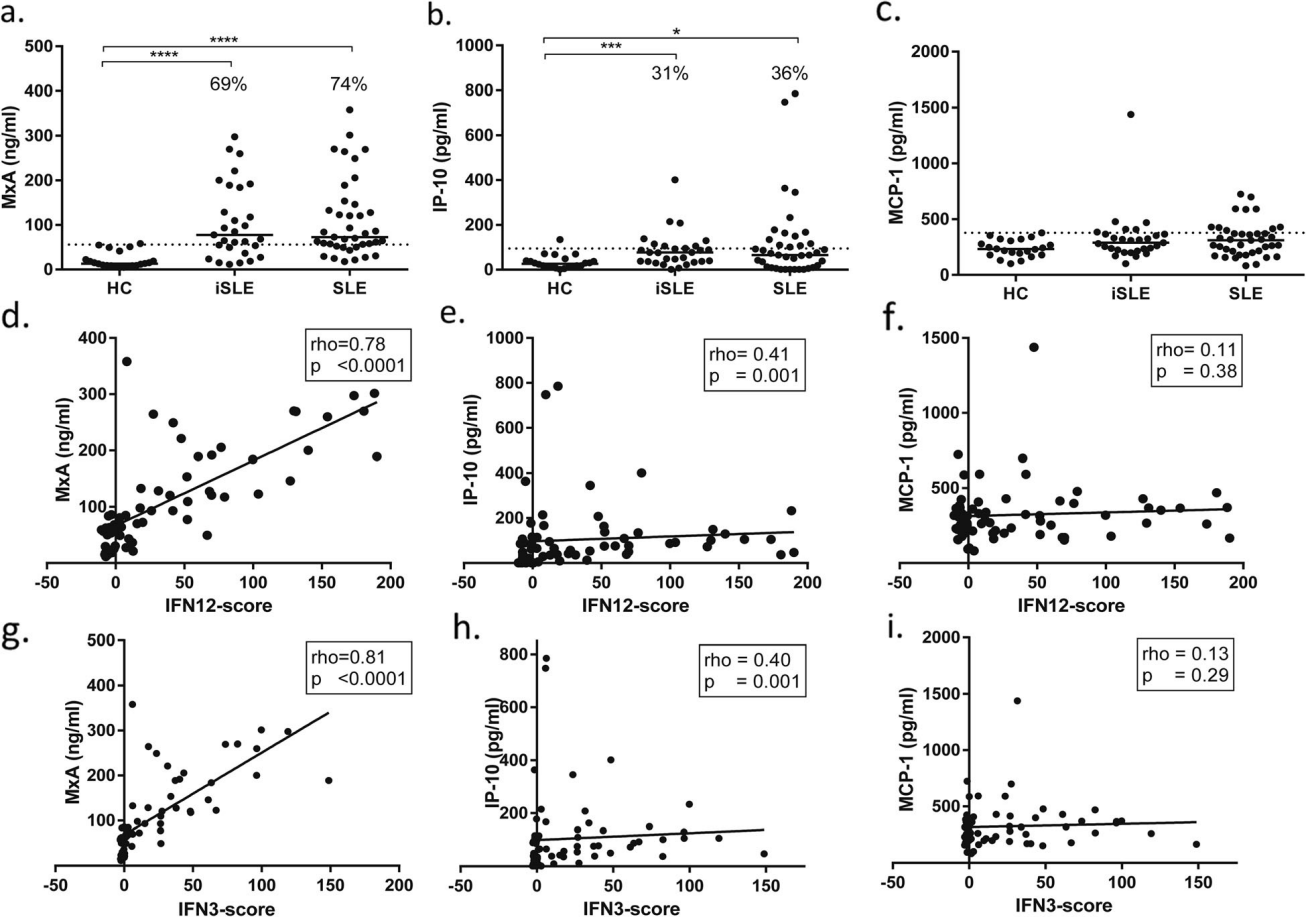
Serum levels of IFN-related soluble mediators MxA (**a**), IP-10 (**b**), and MCP-1 (**c**) in the three groups and their correlations with IFN12-score (**d**–**f**) and IFN3-score (**g**–**i**) in iSLE and SLE patients. The dotted line represents mean + 2SD of the HC. The percentages represent the proportion of patients above this line. Abbreviations: HC healthy controls, iSLE incomplete systemic lupus erythematosus, SLE systemic lupus erythematosus, IFN interferon, MxA myxovirus resistance protein A, IP-10 interferon gamma-induced protein 10, MCP-1 monocyte chemoattractant protein 1

MxA levels were increased in 20 (69%) of iSLE patients (median 77.5 ng/mL) and 29 (74%) of SLE patients (median 72.6 ng/mL) (Fig. 2a). In iSLE, MxA levels correlated positively with ESR (*r* = 0.50, *p* = 0.005), anti-SSA titer (*r* = 0.47, *p* = 0.01), and number of autoantibodies (*r* = 0.47, *p* = 0.01) and negatively with C3 levels (*r* = − 0.42, *p* = 0.02) and C4 (*r* = − 0.40, *p* = 0.03) (see also Additional file 1: Table 3).

IP-10 levels were increased in 9 (31%) of iSLE patients (median 76.9 pg/mL) and 20 (36%) of SLE patients (median 65.7 pg/mL) (Fig. 2b). In iSLE, IP-10 levels correlated with the number of ACR criteria (*r* = 0.38, *p* = 0.04) and SLICC criteria (*r* = 0.37, *p* = 0.05) and negatively with C3 levels (*r* = − 0.37, *p* = 0.05).

MCP-1 levels were increased in 4 (14%) iSLE patients (median 288 pg/mL) and 10 (39%) SLE patients (median 311 pg/mL), but were not significantly higher than HC (Fig. 2c).

### IFN-related soluble mediators—surrogate marker

**Fig. 3:**
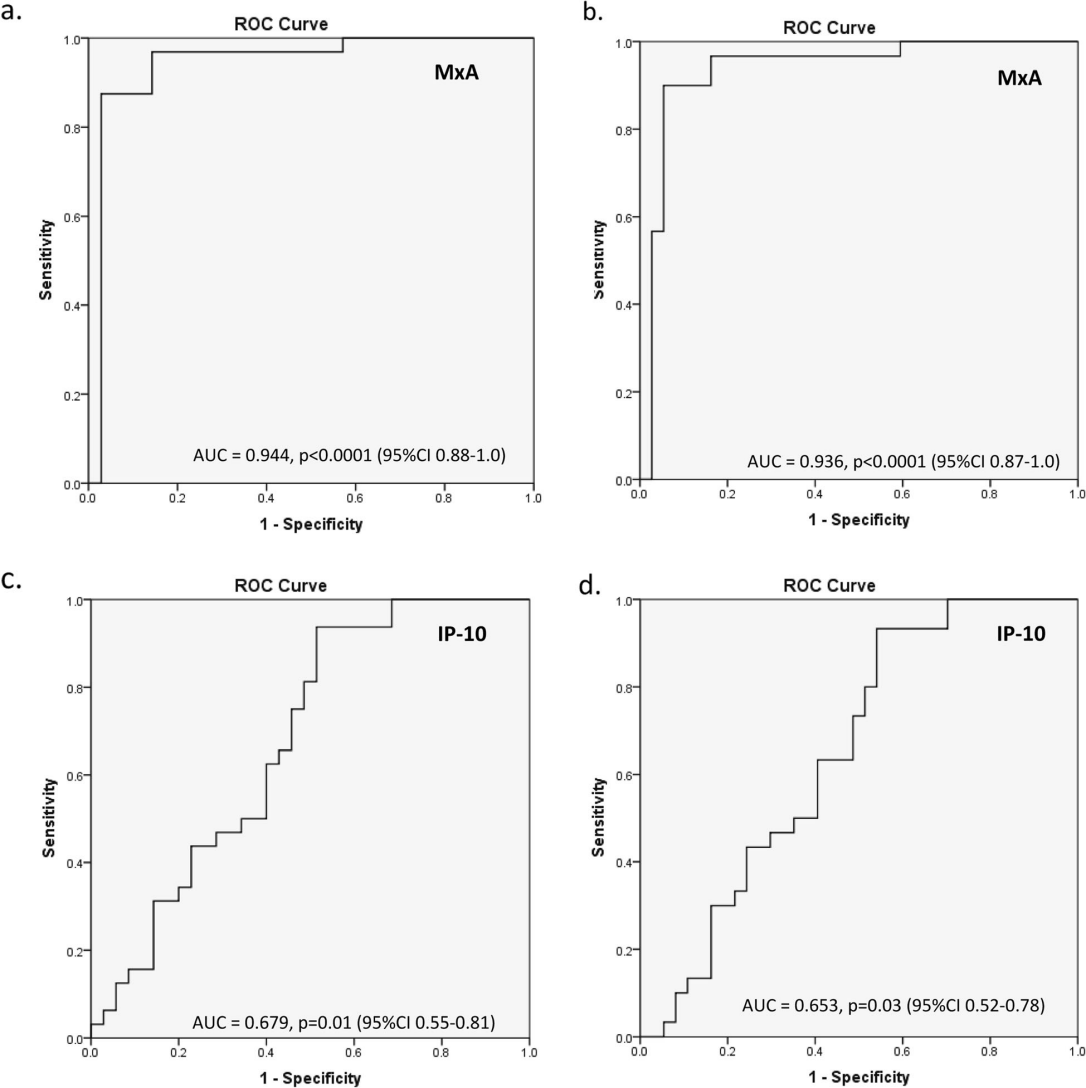
Receiver operator characteristic (ROC) curve of **a** IFN12-score and MxA, **b** IFN3-score and MxA, **c** IFN12-score and IP-10, and **d** IFN3-score and IP-10. Abbreviations: AUC area under the curve, CI confidence interval, MxA myxovirus-resistance protein A, IP-10 interferon gamma-induced protein 10

The relationship between IFN-related soluble mediators and IFN-score was analyzed in the combined patient groups (iSLE and SLE). Interestingly, MxA correlated strongly with IFN12-score (*r* = 0.78, *p* < 0.0001) and IFN3-score (*r* = 0.81, *p* < 0.0001) (Fig. 2d, g). The receiver operator characteristic (ROC) curve for MxA shows an area under the curve (AUC) of 0.94 (95% CI 0.88–1.0) for the IFN12-score and 0.94 (95% CI 0.87–1.0) for the IFN3-score (Fig. 3a, b). IP-10 levels were weakly correlated with the IFN12-score (*r* = 0.39, *p* = 0.001) and with the IFN3 gene-score (*r* = 0.43, *p* = 0.003) (Fig. 2e, h). The ROC curve for IP-10 showed an AUC of 0.68 (95% CI 0.55–0.81) for the IFN12-score and an AUC of 0.65 (95% CI 0.52–0.78) (Fig. 3c, d). MCP-1 levels were not correlated with IFN-scores (Fig. 2f, i).

The tested mediators correlated with each other: MxA and IP-10 (*r* = 0.48, *p* = 0.004), MxA and MCP-1 (*r* = 0.26, *p* = 0.03), and IP-10 and MCP-1 (*r* = 0.35, *p* = 0.004).

### MxA expression in the skin

**Fig. 4:**
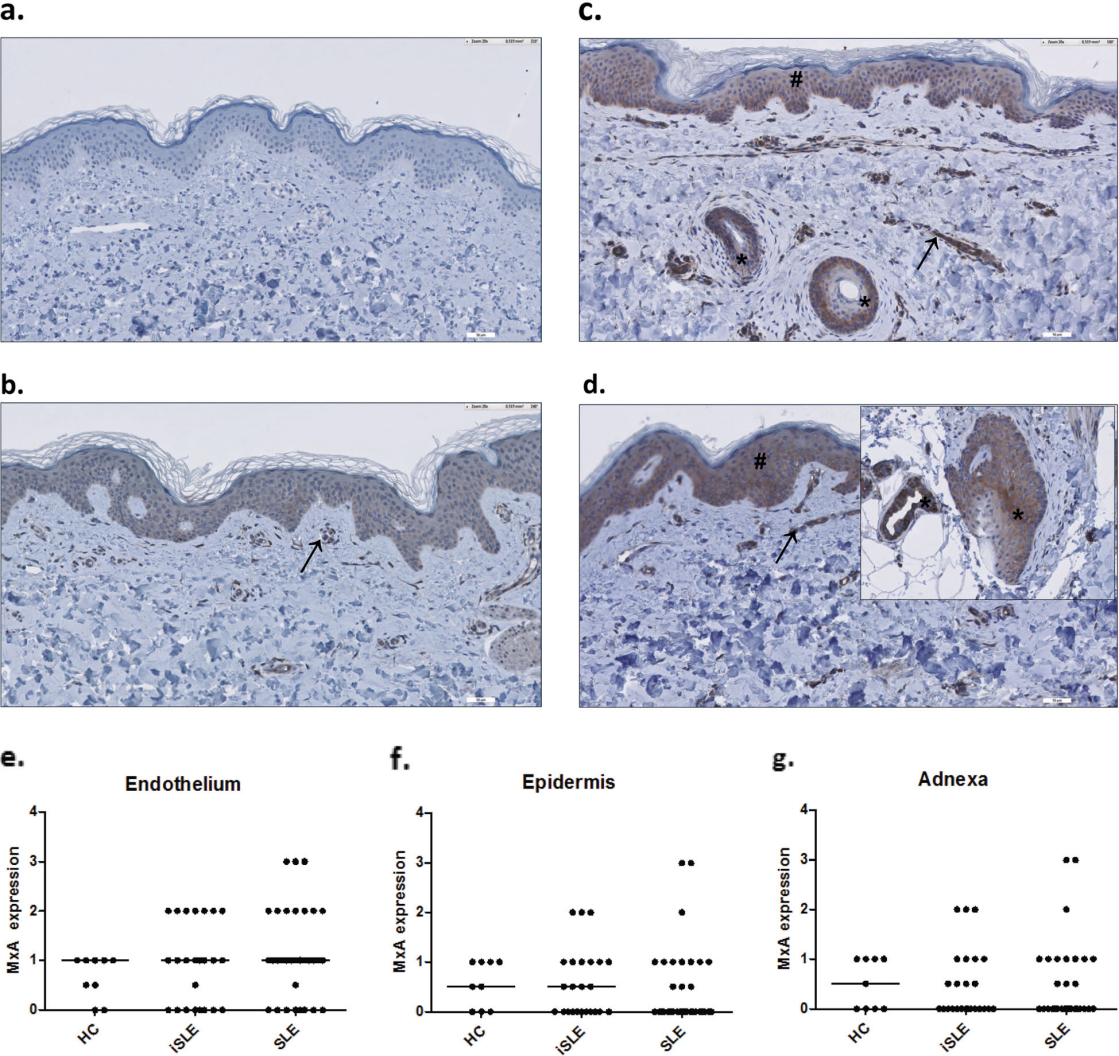
MxA expression in the skin of patients with incomplete systemic lupus erythematosus (iSLE) in comparison with healthy controls (HC) and SLE patients. Representative MxA stainings of a healthy control (**a**), an iSLE patient with positive MxA staining (++) of endothelium only (**b**), and an iSLE patient with positive MxA staining (++) of both the endothelium, the epidermis, and a hair follicle (**c**), and an SLE patient with positive (+++) MxA staining of the endothelium (→), the epidermis (#), a hair follicle, and sweat gland (*) (**d**). Dot plots of semiquantitive MxA expression in the skin of all groups are shown for the endothelium (**e**), epidermis (**f**), and adnexa (**g**)

MxA staining was performed in sections of 9 HC, 24 iSLE patients, and 32 SLE patients. Results are shown in Fig. 4. Some of the HC slides showed moderate MxA expression; therefore, intermediate MxA expression or stronger (score ≥ 2) was regarded positive. MxA expression was most frequently seen in the endothelium and was positive in 7 of 24 (29%) biopsies of iSLE patients and 10 of 32 (31%) of SLE patients. The adnexa and epidermis were positive in 3 of 24 (13%) of iSLE patients and 3 of 32 (9.4%) of SLE patients. These structures were only positive when MxA was also expressed in the endothelium. MxA staining of fibroblasts was positive in only 2 of 24 (8%) iSLE patients and 2 of 32 (6.2%) SLE patients. Infiltrates were almost not present, except in one SLE patient. Because of the limited numbers of positive stainings in the epidermis, adnexa, infiltrates, and fibroblasts, these results were not analyzed.

MxA expression in the skin endothelium of iSLE patients correlated positively with whole blood MxA levels (*r* = 0.53, *p* = 0.008), IFN12-score (*r* = 0.54, *p* = 0.006), IFN3-score (*r* = 0.56, *p* = 0.005), but not with IP-10 serum levels (*r* = 0.37, *p* = 0.073) or MCP-1 serum levels (*r* = 0.21, *p* = 0.34). Additionally, iSLE patients with increased MxA expression in the skin endothelium had increased ANA titers (*p* = 0.02), an increased number of different antibodies (*p* = 0.01), and increased IgG levels (*p* = 0.02). The same correlations were found in SLE (see Additional file 1: Table S4).

## Discussion

Research on iSLE is scarce, but is of great importance in order to better understand the pathophysiology of the disease and to find diagnostic and prognostic biomarkers. IFN type I is an important early mediator in SLE and therefore is worthwhile to investigate in patients with iSLE. In this study, IFN gene expression was increased in 50% of iSLE patients and correlated with ESR, low complement, IgG, and the number of different autoantibody types. As this study was cross-sectional in nature, no conclusions can be drawn about the predictive value of these findings. However, it is known that autoantibody diversity substantially increases around the time point of SLE establishment [28, 29]. Furthermore, decreased complement and higher IgG reactivity were previously reported to be associated with progression to SLE in longitudinal studies on iSLE [10, 30]. Thus, correlation of IFN-score with these factors supports the assumption of a relation with progression to established disease, although longitudinal follow-up is warranted to confirm this.

To our knowledge, three other studies have been published concerning IFN signature in iSLE patients. Recently, Yusof et al. published results on a 12 month follow-up of patients at risk of developing SLE [13]. They included 118 patients with ANA positivity of at least 1:80 titer and not more than 1 clinical SLICC criterion. The researchers calculated a two-score system based on factor analysis, of which IFN score A consists of IFN genes from module 1.2 and module 3.4, whereas IFN score B mainly consists of genes from module 5.12. During the 12 month follow-up, 14 (12%) progressed to SLE and 5 (4%) progressed to primary Sjogren’s disease. The patients who progressed had higher IFN score A and B at baseline. Olsen et al. found increased expression of three IFN-related genes (MX1, OAS1, and IFI27) in iSLE (defined as meeting 1 to 3 ACR criteria), with *p* values around 0.002 compared to healthy controls, which is quite similar to our results [31]. Percentages of IFN-high individuals were not mentioned in this study, and associations between IFN-scores and clinical or serological symptoms were also not reported. Other than in our study, iSLE patients who used hydroxychloroquine had lower IFN-scores than treatment-naïve iSLE patients. Interestingly, this was not the case in SLE patients on hydroxychloroquine. Furthermore, Li et al. found an upregulation of IFN genes in 50% of iSLE patients, which was associated with higher levels of ANA and a number of other autoantibodies, including anti-RNP, anti-dsDNA, and anti-SSA [32]. Our study is in line with these studies, although we did not find a correlation with all mentioned autoantibodies, probably because of limited sample size.

Remarkably, the percentage of SLE patients with an increased IFN12-score in the current study (46%) was lower than that reported in the literature, and even lower than that in iSLE. This is probably attributable to the fact that all patients had quiescent disease. The presence of the IFN signature is associated with cross-sectional disease activity and levels of anti-dsDNA [33]. At the time of blood withdrawal, 15% of the SLE patients had no detectable ANA anymore, and only half of the patients who previously expressed anti-dsDNA still had increased titers of this autoantibody. Also, a negative correlation between IFN expression and time from last disease flare was found in one study, which suggests that IFN signature may be less expressed during disease course [34]. Another explanation for different IFN expression findings may be the lack of standard procedures for determining IFN signature. In literature, different genes, methods, and biological substances have been described for measuring IFN gene expression. In order to improve comparability with previous studies, we calculated an interferon score based on 3 IFN genes (IFI44L, LY6E, and MX1) that are among the most frequently tested in SLE patients [20]. Upon using this score, 51% of SLE patients were positive, which is in line with other studies.

Furthermore, we analyzed genes from all three known interferon-related modules, as published by Chaussabel et al. [19]. In a previous study in SLE patients, only module 1.2 was stable over time, suggesting that this module best reflects the presence of SLE [20]. In the current study, IFN1.2 genes were more often upregulated than IFN3.4 and IFN5.12 genes. Longitudinal analysis will have to point out if distinguishing these modules is of predictive value.

It is important to report that 3 of the 22 healthy controls (14%) had ANA titers ≥ 1:80 (2 subjects had titers of 1:80, nuclear pattern, and coarsely speckled, and 1 subject had a titer of 1:160, coarsely speckled). The values of the IFN12-scores of these subjects were 3.10, − 4.03, and − 4.97. As the IFN-score reflects the number of standard deviations relative to the mean of the control group, only one of these subjects had higher IFN expression than the mean of the healthy controls; however, some ANA-negative healthy controls had higher IFN-scores. Also, when comparing ANA-positive healthy controls with ANA-negative healthy controls, the values of complement and IgG were not clearly different. Therefore, we do not expect that ANA positivity in our control group influenced the outcomes of this study.

Hydroxychloroquine (HCQ) is the backbone of SLE treatment, and its effect is assumed to be mainly based on blockade of TLR7 and 9, which prohibits production of IFN-alpha by pDCs [35]. There is some evidence for delayed onset of SLE after starting treatment with this drug [36]. In the current study, IFN expression levels were not different among patients using HCQ. Our findings are in contrast with the results of Olsen et al. who observed markedly lower IFN gene expression in iSLE patients using HCQ [31]. This difference can possibly be explained by other disease characteristics of the patients on this medication. Ideally, iSLE patients will be longitudinally followed before and after initiation of HCQ, in order to investigate the direct effect on IFN expression.

Analysis of IFN-related genes is time-consuming and therefore not ideal for clinical practice. Therefore, in this study, interferon-related mediators were measured. MxA was already shown to correlate strongly with IFN gene expression levels in patients with Sjögren’s disease and in SLE [37]. In this study, MxA indeed correlated strongly with IFN-scores in both iSLE and SLE, with an impressive AUC of 0.94, and also correlated with the same clinical and serological parameters. MxA thus indeed appears to be a suitable and accurate surrogate marker for IFN type I gene expression.

Also, two other IFN-related chemokines were measured in iSLE and linked to clinical and serological features. These chemokines, other than MxA, which should be analyzed in whole blood, were tested in serum. Levels of IP-10-which are induced by IFN-gamma—a type II interferon—have previously been shown to be increased in SLE patients and to correspond longitudinally with disease flares [38]. This chemokine is also of interest as it appeared to be significantly increased in serum of SLE patients compared to pre-symptomatic individuals [22, 39, 40]. In the current study, IP-10 was increased in approximately one third of iSLE patients and corresponded with the number of classification criteria and hypocomplementemia. Furthermore, IP-10 levels correlated with the IFN12-score, but not as strong as MxA. However, the role of this chemokine in the pathogenesis of SLE is not completely understood and longitudinal results should be awaited to validate its predictive value for SLE progression. MCP-1 was not increased in iSLE, nor in SLE, neither did it correspond with disease features. MCP-1 hence does not seem to be a suitable biomarker in iSLE.

To test IFN expression in the skin, MxA staining was performed in unaffected, non-sun-exposed skin biopsies. MxA is strongly expressed in cutaneous lupus lesions and was previously shown to co-localize with effector lymphocytes [41]. Also, the unaffected SLE skin has been shown to express MxA [42]. In the current study, approximately one third of iSLE and SLE patients expressed MxA in skin endothelium, which correlated with whole blood MxA levels and IFN-scores. Only a small percentage (10%) showed positive MxA staining of the epidermis and adnexa, but this is still a remarkable finding given the fact that it concerns the unaffected skin. Interestingly, these structures were only positive when MxA was also expressed in the endothelium, which could imply that circulating autoantigens initially activate IFN production in the skin endothelium and not in the epidermis itself. The actual clinical relevance of MxA expression in unaffected skin cannot be elucidated by our findings. Larger sample sizes and longitudinal follow-up could shed more light on this subject.

There are some limitations to this study. First, most patients were Caucasian, and thus, the results might not be applicable for other ethnic populations. Also, sample size was limited, mainly due to low prevalence of lupus in general and the strict inclusion criteria for iSLE.

## Conclusions

In summary, IFN upregulation is present in half the patients with iSLE and correlates with decreased complement, IgG, and autoantibody diversity. These serological features are all associated with increased risk of developing more severe disease in SLE patients. IFN-induced MxA was found to be expressed in the unaffected skin of approximately one third of iSLE patients and was associated with the same serological features. These findings provide further evidence that iSLE patients with increased IFN type I expression could be at higher risk for developing complete SLE. Longitudinal data, however, should confirm this hypothesis.

Finally, whole blood MxA levels correlated strongly with IFN-scores and could be a suitable and easily applicable surrogate marker for IFN gene expression.

## Supplementary information

**Additional file 1: Figure S1.** Control stainings. a. Positive control: MxA expression in lesional lupus (chronic discoid lupus erythematosus)skin. b. Negative control: staining with normal goat serum and conjugate. c. Negative control: staining with PBS and conjugate. **Table S1.** Correlation matrix of IFN12-score and IFN3-score with continuous variables. **Table S2.** Correlation matrix of MxA and IP-10. **Table S3.** Correlations of MxA-expression in skin endothelium with serological parameters. **Figure S2.** Significant correlations of IFN12-score with (a) ESR, (b) Monocytes, (c) Number of autoantibodies, (d) anti-SSA titer, (e) C4, and (f) IgG.

## Data Availability

The datasets during and/or analyzed during the current study are available from the corresponding author on reasonable request.

## Abbreviations

ACR: American College of Rheumatology
ANA: Antinuclear antibody
Anti-dsDNA: Anti-double-stranded DNA
DAB: Diaminobenzidine
ELISA: Enzyme-linked immunosorbent assay
HC: Healthy control
HCQ: Hydroxychloroquine
IFN: Interferon
IgG: Immunoglobulin G
IP-10: Interferon-γ-induced protein 10
iSLE: Incomplete systemic lupus erythematosus
MCP-1: Monocyte chemoattractant protein 1
MxA: Myxovirus resistance protein A
pDC: Plasmocytoid dendritic cell
RE: Relative expression
RT-PCR: Real-time polymerase chain reaction
SLE: Systemic lupus erythematosus
SLEDAI: SLE Daily Activity Index
SLICC: Systemic Lupus International Collaborating Clinics
TLR: Toll-like receptor
