# Supplementary material for: Interferon score is increased in incomplete systemic lupus erythematosus and correlates with myxovirus-resistance protein A in blood and skin
Source: Arthritis Res Ther. 2019 Dec 2;21:260. doi: 10.1186/s13075-019-2034-4 (PMC6889676; doi:10.1186/s13075-019-2034-4)

**Supplementary figure 1. Control stainings.**

**a. Positive control: MxA expression in lesional lupus (chronic discoid lupus erythematosus)**

**skin.**

**b. Negative control: staining with normal goat serum and conjugate.**

**c. Negative control: staining with PBS and conjugate.**


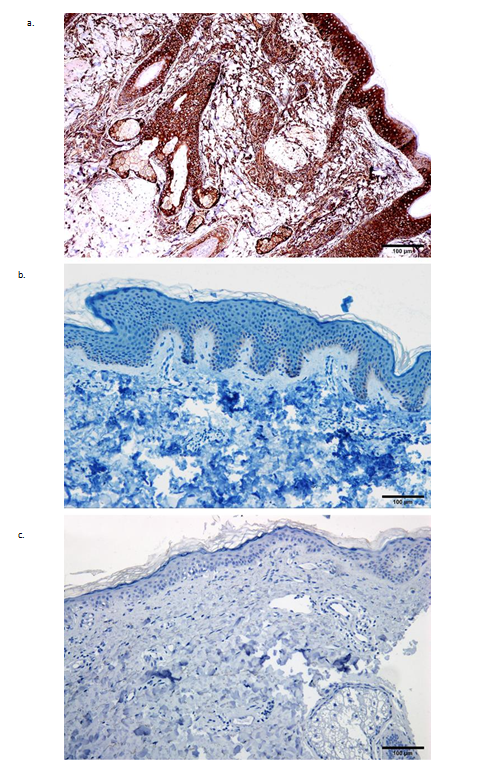


**Supplementary table 1. Correlation matrix of IFN12-score and IFN3-score with continuous variables**

|  | **iSLE** | | | | **SLE** | | | | |
| --- | --- | --- | --- | --- | --- | --- | --- | --- | --- |
|  | **IFN12-score** | | **IFN3-score** | | **IFN12-score** | | **IFN3-score** | | |
|  | *Rho* | *p* | *Rho* | *p* | *Rho* | *P* | *Rho* | *P* | |
| **Age** | -0.32 | 0.10 | -0.32 | 0.05 | -0.12 | 0.47 | -0.08 | 0.61 | |
| **Disease duration** | -0.04 | 0.83 | -0.05 | 0.79 | -0.03 | 0.86 | -0.01 | 0.95 | |
| **ACR criteria** | 0.09 | 0.66 | 0.07 | 0.71 | -0.03 | 0.85 | -0.02 | 0.90 | |
| **SLICC criteria** | 0.17 | 0.40 | 0.11 | 0.59 | 0.01 | 0.94 | 0.003 | 0.99 | |
| **SLEDAI** | 0.22 | 0.25 | 0.22 | 0.27 | **0.35** | **0.03** | 0.42 | 0.007 | |
| **ESR** | **0.45** | **0.02** | **0.48** | **0.01** | 0.28 | 0.10 | 0.28 | 0.10 | |
| **Hb** | -0.30 | 0.12 | -0.28 | 0.15 | 0.009 | 0.10 | 0.02 | 0.89 | |
| **Leukocytes** | **-0.42** | **0.03** | -0.35 | 0.07 | **-0.33** | **0.04** | **-0.33** | **0.04** | |
| **Lymphocytes** | -0.21 | 0.28 | -0.16 | 0.43 | 0.07 | 0.67 | 0.05 | 0.75 | |
| **Monocytes** | -0.37 | 0.05 | -0.29 | 0.13 | -0.04 | 0.82 | -0.04 | 0.82 | |
| **Neutrophils** | **-0.39** | **0.04** | -0.33 | 0.09 | **-0.40** | **0.01** | **-0.39** | **0.02** | |
| **Thrombocytes** | -0.20 | 0.32 | -0.17 | 0.38 | -0.22 | 0.19 | -0.20 | 0.23 | |
| **CRP** | 0.11 | 0.57 | 0.08 | 0.69 | -0.006 | 0.97 | -0.03 | 0.87 | |
| **GFR** | 0.31 | 0.11 | 0.25 | 0.20 | 0.15 | 0.38 | 0.10 | 0.57 | |
| **ANA titer** | 0.27 | 0.19 | 0.30 | 0.14 | 0.23 | 0.16 | 0.25 | 0.13 | |
| **Anti-dsDNA** | -0.32 | 0.10 | -0.28 | 0.15 | 0.24 | 0.15 | 0.25 | | 0.13 |
| **Anti-SSA** | **0.45** | **0.02** | **0.47** | **0.01** | **0.33** | **0.04** | **0.35** | | **0.03** |
| **Nr of AutoAb** | **0.52** | **0.005** | **0.56** | **0.002** | **0.46** | **0.004** | **0.51** | | **0.001** |
| **C3** | -0.37 | 0.053 | **-0.39** | **0.04** | **-0.33** | **0.04** | **-0.35** | | **0.03** |
| **C4** | **-0.49** | **0.008** | **-0.44** | **0.02** | -0.20 | 0.23 | 0.21 | | 0.21 |
| **IgG** | 0.37 | 0.06 | **0.41** | **0.04** | 0.20 | 0.22 | 0.17 | | 0.31 |

Abbreviations: ANA = antinuclear antibody, Anti-dsDNA = anti-doublestranded DNA, ESR = erythrocyte sedimentation rate

| **iSLE** | | | | | | |
| --- | --- | --- | --- | --- | --- | --- |
|  | **MxA** | | **IP-10** | | **MCP-1** | |
|  | *Rho* | *p* | *Rho* | *p* | *Rho* | *P* |
| **Age** | -0.11 | 0.56 | -0.03 | 0.90 | 0.26 | 0.17 |
| **Disease duration** | -0.13 | 0.50 | -0.07 | 0.72 | -0.18 | 0.36 |
| **ACR criteria** | 0.14 | 0.47 | **0.38** | **0.04** | -0.05 | 0.81 |
| **SLICC criteria** | 0.12 | 0.55 | **0.41** | **0.03** | 0.05 | 0.81 |
| **SLEDAI** | 0.21 | 0.27 | 0.60 | 0.001 | -0.02 | 0.91 |
| **ESR** | **0.50** | **0.005** | 0.15 | 0.44 | -0.15 | 0.43 |
| **Hb** | -0.25 | 0.19 | 0.08 | 0.70 | 0.21 | 0.27 |
| **Leukocytes** | -0.32 | 0.09 | -0.29 | 0.13 | -0.06 | 0.74 |
| **Lymphocytes** | -0.22 | 0.25 | -0.30 | 0.12 | -0.09 | 0.65 |
| **Monocytes** | -0.20 | 0.30 | 0.04 | 0.84 | 0.16 | 0.42 |
| **Neutrophils** | -0.28 | 0.14 | -0.26 | 0.17 | -0.18 | 0.36 |
| **Thrombocytes** | -0.18 | 0.34 | -0.34 | 0.07 | -0.19 | 0.32 |
| **CRP** | 0.07 | 0.72 | 0.12 | 0.54 | 0.15 | 0.43 |
| **GFR** | 0.19 | 0.33 | 0.07 | 0.71 | 0.33 | 0.08 |
| **ANA titer** | 0.32 | 0.11 | 0.16 | 0.45 | 0.07 | 0.74 |
| **Anti-dsDNA** | -0.20 | 0.31 | 0.14 | 0.45 | **-0.38** | **0.04** |
| **Anti-SSA** | **0.47** | **0.01** | 0.17 | 0.37 | 0.30 | 0.12 |
| **Nr of AutoAb** | **0.65** | **<0.0001** | 0.36 | 0.05 | 0.26 | 0.17 |
| **C3** | **-0.41** | **0.03** | **-0.37** | **0.05** | 0.06 | 0.77 |
| **C4** | **-0.38** | **0.04** | -0.34 | 0.07 | -0.04 | 0.86 |
| **IgG** | 0.32 | 0.10 | 0.35 | 0.07 | -0.27 | 0.16 |

**Supplementary table 2. Correlation matrix of MxA and IP-10**

Abbreviations: ANA = antinuclear antibody, Anti-dsDNA = anti-doublestranded DNA, ESR = erythrocyte sedimentation rate

| **SLE** | | | | | | |
| --- | --- | --- | --- | --- | --- | --- |
|  | **MxA** | | **IP-10** | | **MCP-1** | |
|  | *Rho* | *p* | *Rho* | *p* | *Rho* | *P* |
| **Age** | -0.08 | 0.63 | 0.14 | 0.38 | -0.07 | 0.69 |
| **Disease duration** | **0.33** | **0.04** | -0.10 | 0.56 | 0.12 | 0.47 |
| **ACR criteria** | -0.06 | 0.73 | 0.07 | 0.66 | -0.02 | 0.89 |
| **SLICC criteria** | -0.08 | 0.63 | -0.16 | 0.34 | -0.22 | 0.19 |
| **SLEDAI** | 0.39 | 0.02 | 0.10 | 0.53 | 0.08 | 0.62 |
| **ESR** | 0.09 | 0.62 | **0.55** | **0.001** | 0.16 | 0.34 |
| **Hb** | 0.004 | 0.98 | -0.21 | 0.19 | -0.08 | 0.64 |
| **Leukocytes** | -0.03 | 0.86 | **-0.56** | **<0.0001** | -0.02 | 0.91 |
| **Lymphocytes** | 0.05 | 0.78 | -0.14 | 0.41 | -0.07 | 0.70 |
| **Monocytes** | 0.16 | 0.35 | -0.06 | 0.70 | -.002 | 0.1 |
| **Neutrophils** | -0.02 | 0.91 | **-0.61** | **<0.0001** | -0.07 | 0.68 |
| **Thrombocytes** | -0.14 | 0.39 | **-0.35** | **0.03** | -0.15 | 0.38 |
| **CRP** | -0.11 | 0.51 | **0.36** | **0.02** | 0.08 | .062 |
| **GFR** | 0.09 | 0.58 | 0.18 | 0.28 | -0.10 | 0.95 |
| **ANA titer** | 0.09 | 0.59 | **0.37** | **0.02** | **0.33** | **0.04** |
| **Anti-dsDNA** | 0.05 | 0.77 | 0.16 | 0.33 | 0.04 | 0.82 |
| **Anti-SSA** | **0.37** | **0.02** | 0.23 | 0.16 | **0.35** | **0.03** |
| **Nr of AutoAb** | **0.57** | **<0.0001** | **0.49** | **0.001** | **0.43** | **0.006** |
| **C3** | -0.20 | 0.23 | -0.04 | 0.80 | 0.01 | 0.94 |
| **C4** | -0.07 | 0.68 | -0.06 | 0.72 | 0.10 | 0.53 |
| **IgG** | 0.002 | 0.99 | **0.37** | **0.02** | -0.13 | 0.42 |

**Supplementary Table 3. Correlations of MxA-expression in skin endothelium with serological parameters**

|  | **iSLE** | | **SLE** | | **iSLE+SLE** | |
| --- | --- | --- | --- | --- | --- | --- |
|  | *Rho* | *p* | *Rho* | *p* | *Rho* | *p* |
| **MxA** | **0.53** | **0.008** | **0.62** | **<0.0001** | **0.59** | **<0.0001** |
| **IFN12-score** | **0.54** | **0.006** | **0.54** | **0.002** | **0.54** | **<0.0001** |
| **IFN3-score** | **0.59** | **0.003** | **0.54** | **0.001** | **0.57** | **<0.0001** |
| **IP-10** | 0.37 | 0.07 | 0.32 | 0.08 | **0.33** | **0.01** |
| **C3** | -0.32 | 0.13 | -0.19 | 0.30 | **-0.27** | **0.04** |
| **C4** | -0.24 | 0.25 | -0.22 | 0.23 | -0.24 | 0.07 |
| **Autoantibody number** | **0.56** | **0.004** | 0.18 | 0.33 | **0.32** | **0.02** |
| **Anti-dsDNA** | 0.03 | 0.91 | 0.06 | 0.76 | 0.09 | 0.51 |
| **IgG** | **0.46** | **0.03** | **0.39** | **0.03** | **0.37** | **0.005** |

Abbreviations: MxA = myxovirus resistance protein A, IFN = interferon, IP-10 = interferon-gamma induced protein 10, C = complement, Anti-dsDNA = anti-doublestranded DNA

**Supplementary Figure 2.** Significant correlations of IFN12-score with (a) ESR, (b) Monocytes, (c) Number of autoantibodies, (d) anti-SSA titer, (e) C4, and (f) IgG


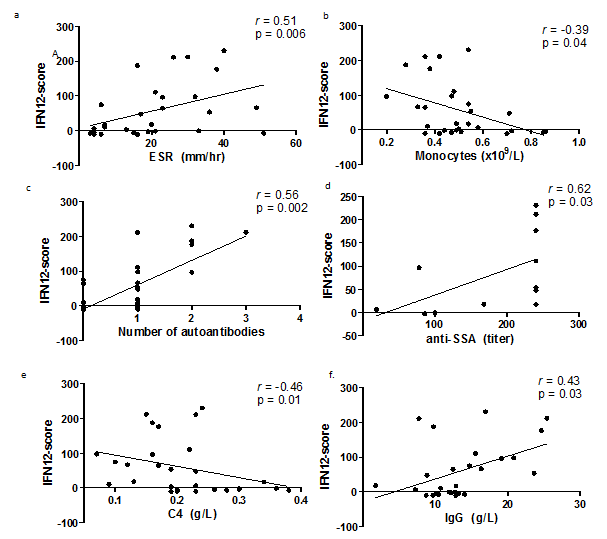

Supplement: Supplementary file 1 — Additional file 1: Figure S1. Control stainings. a. Positive control: MxA expression in lesional lupus (chronic discoid lupus erythematosus)skin. b. Negative control: staining with normal goat serum and conjugate. c. Negative control: staining with PBS and conjugate. Table S1. Correlation matrix of IFN12-score and IFN3-score with continuous variables. Table S2. Correlation matrix of MxA and IP-10. Table S3. Correlations of MxA-expression in skin endothelium with serological parameters. Figure S2. Significant correlations of IFN12-score with (a) ESR, (b) Monocytes, (c) Number of autoantibodies, (d) anti-SSA titer, (e) C4, and (f) IgG. [file 13075_2019_2034_MOESM1_ESM.docx]
